# Successful Endovascular Retrieval of a Partially Disrupted Catheter Using a Crossing Catheter: A Bailout Technique for Severely Calcified Peripheral Lesions

**DOI:** 10.3400/avd.cr.26-00089

**Published:** 2026-09-29

**Authors:** Keisuke Oshima, Shigeo Ichihashi, Yuto Chanoki, Shinichi Iwakoshi, Takahiro Nakai, Sho Shimizu, Terunari Okuyama, Toshihiro Tanaka

**Affiliations:** Department of Diagnostic and Interventional Radiology, Nara Medical University, Kashihara, Nara, Japan

**Keywords:** lower extremity artery disease, endovascular treatment, bailout procedure

## Abstract

Catheter disruption is a rare but potentially serious complication that may occur during catheter retrieval after passage through a heavily calcified lesion, particularly when the catheter becomes entrapped within the calcified plaque. Prompt retrieval is essential to avoid complete disruption of the catheter, thrombosis, or the need for surgical removal. We report 2 cases in which a partially disrupted catheter was successfully retrieved using a crossing catheter as a bailout technique. This approach may be useful for managing catheter entrapment and impending catheter fracture in severely calcified lesions.

## Introduction

Endovascular treatment (EVT) has become widely adopted for patients with lower extremity artery disease. However, EVT in patients with chronic limb-threatening ischemia remains technically challenging because these patients often present with highly complex lesions characterized by severe calcification, chronic total occlusion, and long lesions.^1)^ In such heavily calcified lesions, catheters and other devices may encounter substantial resistance during advancement or retrieval because of severe luminal narrowing, rigid calcified plaque, and device entrapment. Although device delivery failure is relatively common in this setting, catheter entrapment with partial disruption is rare but potentially serious.^2,3)^ When a catheter becomes stuck within a heavily calcified lesion, forceful retraction may cause structural damage to the catheter, resulting in partial disruption or impending complete fracture. Complete catheter fracture or intravascular retention may require endovascular retrieval or even surgical removal; therefore, prompt bailout management is essential.^4,5)^ However, there are limited reports describing practical endovascular techniques for retrieving a partially disrupted catheter before complete fracture occurs. The Wingman 35 crossing catheter (Reflow Medical, San Clemente, CA, USA) is designed to facilitate the crossing of resistant lesions. In addition to its crossing capability, its beveled tip may facilitate bailout retrieval by enabling coaxial advancement through severely calcified plaque and localized plaque modification around the entrapped device. Herein, we report 2 cases of successful retrieval of a partially disrupted catheter using a Wingman 35 after catheter entrapment within a severely calcified lesion.

## Case Report

### Case 1

A woman in her 70s with end-stage renal disease on hemodialysis was referred to our department with bilateral intermittent claudication, left rest pain, and an ulcer on the left heel. Her medical history included percutaneous coronary intervention for coronary artery disease, hypertension, diabetes mellitus, and dyslipidemia. The ankle–brachial index (ABI) was unmeasurable bilaterally, and the skin perfusion pressure (SPP) on the left foot was 14 mmHg at the dorsum and 32 mmHg at the plantar aspect. Computed tomography angiography revealed diffuse, heavily calcified stenoses extending from the bilateral superficial femoral arteries (SFAs) to the popliteal arteries (PAs).

Because of the ischemic ulcer, EVT was first performed for the left lower extremity. Angiography demonstrated diffuse stenosis from the SFA to the PA, with a severely calcified stenosis at the P1–2 segment of the PA. A Parent Plus 45 guiding sheath (Medikit, Tokyo, Japan) was positioned just proximal to the target lesion. Using a 4-Fr diagnostic catheter for backup support, a Teleport XT support catheter (OrbusNeich Medical, Hong Kong, China) and a 0.014-inch Gladius microguidewire (Asahi Intecc, Aichi, Japan) were successfully advanced across the lesion. The guidewire was then exchanged for a VASSALLO GT Support microguidewire (Asahi Intecc).

During attempted withdrawal, the Teleport XT became entrapped within the heavily calcified plaque. Forceful retraction caused unraveling and elongation of the inner coil of the Teleport XT, resulting in narrowing of its inner lumen and firm locking between the Teleport XT and the VASSALLO GT Support microguidewire. The PIERCE technique,^6)^ which consists of percutaneous direct needle puncture of the calcified plaque to create cracks in the severe calcification, was attempted but failed to release the entrapped system.

As a bailout strategy, the hub of the Teleport XT was cut with a surgical scalpel, and a Wingman 35 crossing catheter (Reflow Medical) was advanced coaxially over the locked system. Because the Wingman 35 has a 0.035-inch inner lumen, it could accommodate the outer diameter of the Teleport XT. While continuous traction was applied to the locked system, the Wingman 35 was advanced with a drilling motion through the calcified lesion, allowing successful retrieval of the entrapped catheter and guidewire as a single unit (**Supplementary Video 1**). Post-retrieval angiography showed no obvious vascular injury. The lesion was subsequently rewired, and balloon angioplasty was performed using a 5.0-mm balloon (JADE 5/40 mm; OrbusNeich Medical). Final angiography demonstrated improved stenosis without complications (**Fig. 1**). The postoperative ABI on the left side improved to 0.60, and the patient was discharged uneventfully.

**Fig. 1:**
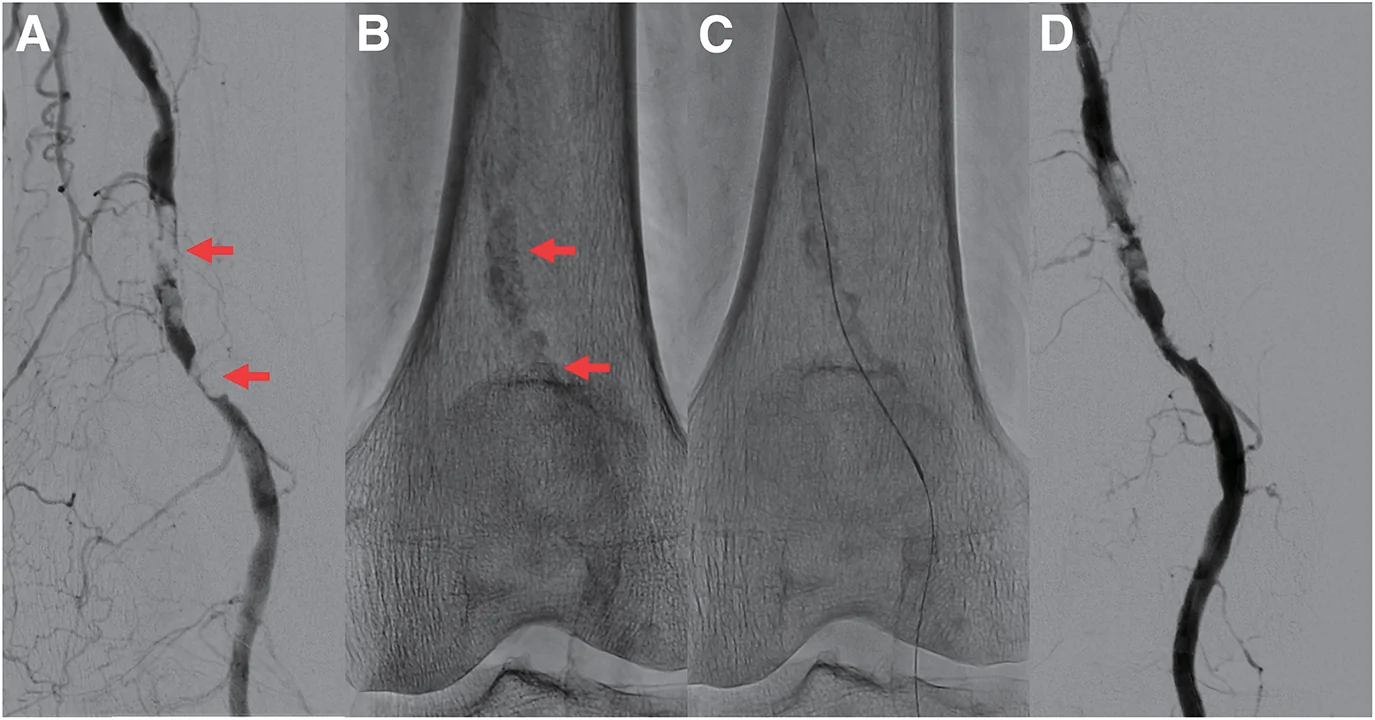
Fluoroscopic findings in Case 1. Red arrows indicate the severely calcified stenosis at the P1–2 segment of the popliteal artery, where the microcatheter system became entrapped. (**A**) Baseline angiography demonstrates diffuse calcified stenosis (arrows). (**B**) Baseline fluoroscopy highlights dense calcification at the target lesion (arrows). (**C**) The entrapped, locked system, consisting of the partially disrupted Teleport XT microcatheter and microguidewire, is shown within the rigid calcified plaque. (**D**) Final angiography shows improved vessel patency without vascular injury.

### Case 2

A man in his 80s with end-stage renal disease on hemodialysis was referred to our department for refractory bilateral lower leg ulcers. His medical history included prior percutaneous coronary intervention for coronary artery disease, cerebral infarction, and hypertension. The ABI was unmeasurable because of severe ulcer-related pain. The SPP was 17 mmHg at the right dorsum, 38 mmHg at the right plantar aspect, 33 mmHg at the left dorsum, and 19 mmHg at the left plantar aspect. Computed tomography angiography revealed diffuse calcified stenoses and occlusions extending from the bilateral SFAs to the PAs.

EVT was performed via antegrade puncture of the left common femoral artery under ultrasound guidance. A 6-Fr, 11-cm sheath was inserted. Angiography demonstrated diffuse stenosis from the SFA to the PA, including severely calcified stenosis from the distal SFA to the P1 segment of the PA and total occlusion of the P3 segment. A Teleport XT support catheter was inserted coaxially through a 4-Fr diagnostic catheter, and several 0.014-inch microguidewires, including Gladius, Halberd, and CROSSLEAD Penetration guidewires (Asahi Intecc), were used sequentially. Although the system successfully crossed the heavily calcified stenosis and reached the P1 segment, the Teleport XT failed to track across the lesion. After unsuccessful predilation with a 3.0-mm balloon, lesion modification was performed at the P1 segment using a Crosser IQ catheter (Becton, Dickinson and Company, Franklin Lakes, NJ, USA).

To obtain stronger backup support for recanalization of the P3 occlusion, a 5.5-Fr GoGo guiding catheter (Medikit) was advanced into the P1 segment of the PA using a 3.0-mm balloon anchor technique (Coyote ES 3 mm/4 cm; Boston Scientific, Marlborough, MA, USA). A 4-Fr diagnostic catheter, Teleport XT support catheter, and Halberd guidewire were then inserted coaxially. With diagnostic catheter support, the Teleport XT and Halberd were successfully advanced across the P3 occlusion.

During attempted withdrawal, the Teleport XT became entrapped in the severely calcified P3 lesion, and forceful retraction caused unraveling and elongation of its inner coil. Similar to Case 1, the hub of the Teleport XT was cut with a scalpel, and a Wingman 35 crossing catheter was advanced coaxially over the disrupted system. By drilling through the calcified plaque, the Wingman 35 was advanced close to the tip of the Teleport XT, enabling the Teleport XT and guidewire to be pulled into the Wingman 35 and retrieved together (**Supplementary Video 2**). Post-retrieval angiography showed no obvious vascular injury.

After successful device retrieval, the lesion was rewired with a Halberd guidewire. Balloon angioplasty was performed using a 5.0-mm Sterling balloon (5 mm/15 cm; Boston Scientific) for the SFA–PA segment and a 3.0-mm JADE balloon (3.0/120 mm; OrbusNeich Medical) for the tibioperoneal trunk and peroneal artery. Final angiography confirmed improved flow through the treated stenotic and occluded segments (**Fig. 2**). The postoperative left ABI improved to 0.94, and the patient was discharged without complications.

**Fig. 2:**
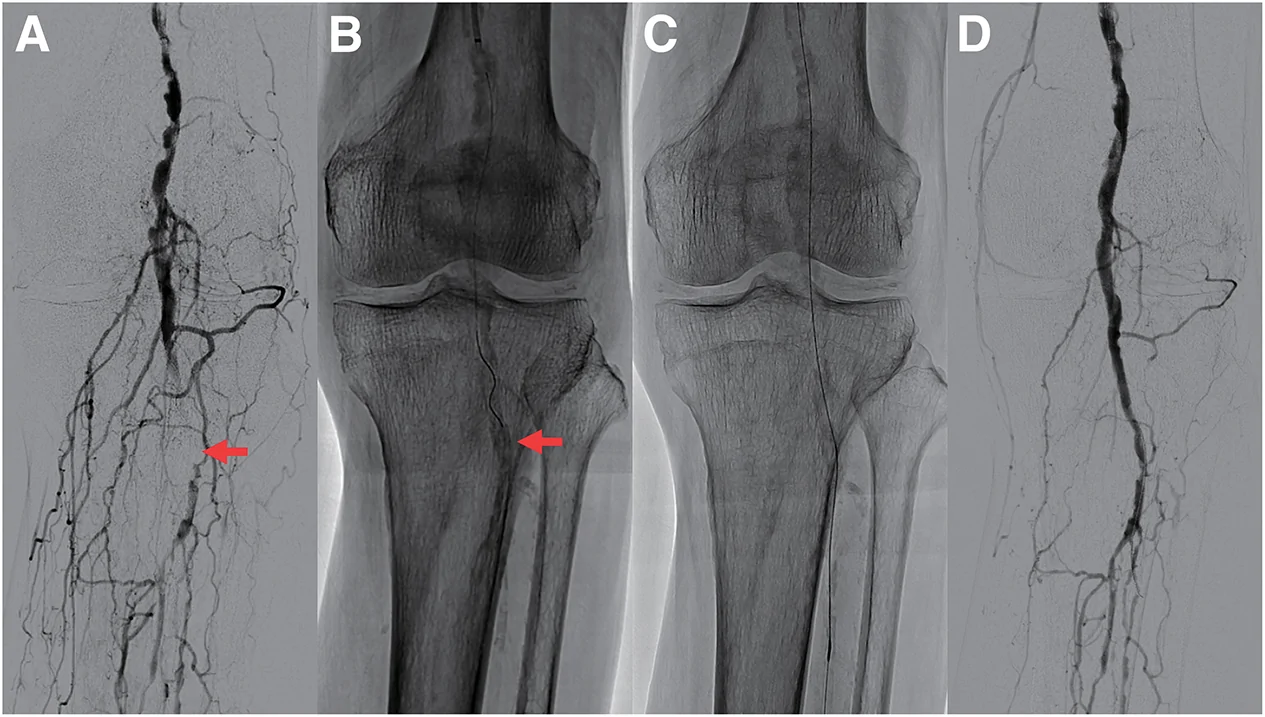
Fluoroscopic findings in Case 2. Red arrows indicate the calcified total occlusion at the P3 segment of the popliteal artery, where the microcatheter system became entrapped. (**A**) Baseline angiography demonstrates the total occlusion with severe calcification (arrow). (**B**) Baseline fluoroscopy highlights the dense calcification at the target lesion (arrow). (**C**) The entrapped, locked system, consisting of the partially disrupted Teleport XT microcatheter and microguidewire, is shown within the rigid calcified plaque. (**D**) Final angiography shows improved vessel patency without vascular injury.

## Discussion

Device entrapment is a well-recognized complication in PCI (e.g., PROGRESS-CTO registry)^4)^ and is increasingly recognized in EVT due to the prevalence of severe, diffuse calcifications in peripheral arteries.^2,3)^ Structurally, when a microcatheter with an inner coil (like the Teleport XT) becomes wedged in dense calcium, forceful manual traction causes the proximal shaft to stretch while the distal tip remains fixed. This longitudinal stretching unravels the coil, narrowing the inner lumen through a finger-trap mechanism and tightly locking the microcatheter and guidewire together as a single unit (**Fig. 3**). Forceful withdrawal significantly increases the risk of device fracture.^4,5)^

**Fig. 3:**
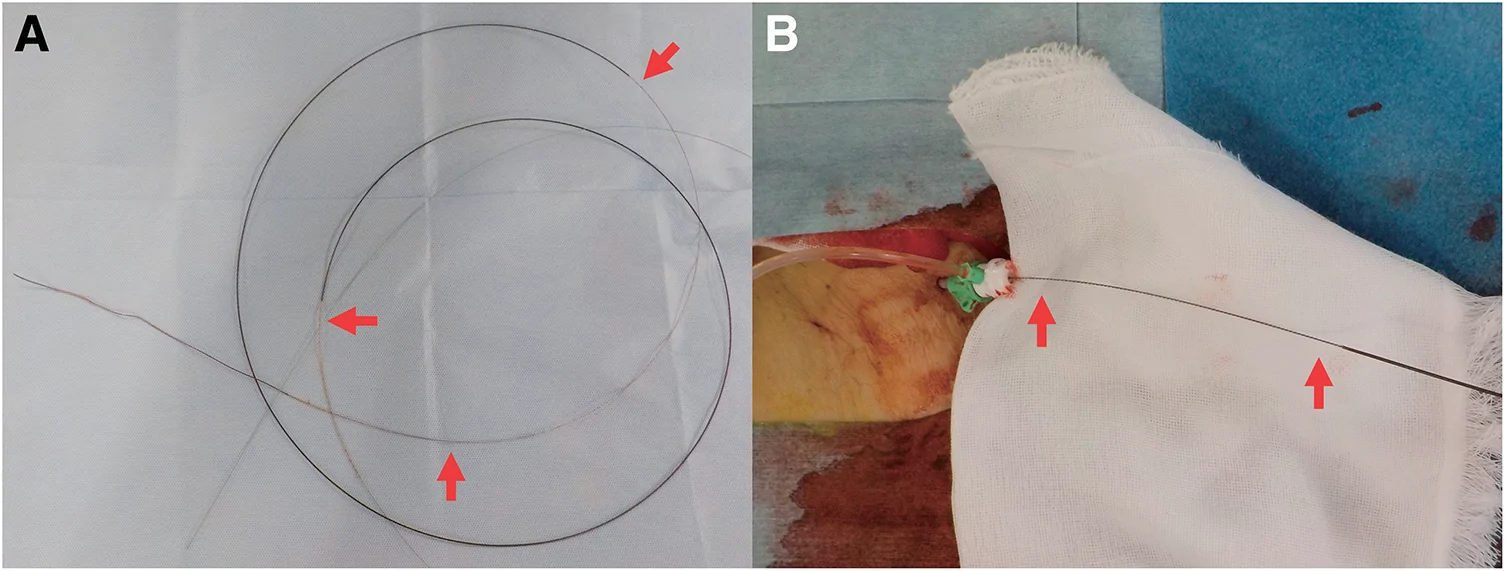
Macroscopic findings of the retrieved, partially disrupted Teleport XT microcatheters. The segments in which the inner coil unraveled and stretched appear lighter in color and visibly narrowed (arrows). (**A**) Case 1. (**B**) Case 2.

Existing bailout techniques have significant limitations in severely calcified lesions. The double-guide catheter technique involves inserting a second sheath adjacent to the initial access site to deliver additional devices, such as a balloon, alongside the entrapped catheter.^2)^ Although useful, this strategy requires sufficient remaining luminal space to advance a parallel device. Similarly, conventional telescoping may not be feasible when rigid calcified plaque prevents further catheter advancement, while retrograde approaches^3)^ carry additional risks of puncture-site complications, particularly in patients with diffuse calcification or ischemic ulcers. Before using the Wingman 35 crossing catheter, we attempted retrieval with a 4-Fr diagnostic catheter advanced to the calcified lesion, but it was unsuccessful. We also attempted the PIERCE technique to release the entrapped Teleport XT support catheter. However, catheter withdrawal remained difficult despite percutaneous puncture of the calcified plaque. One possible explanation is that, in diffuse calcified disease, the exact portion of the calcified plaque responsible for catheter entrapment is difficult to identify fluoroscopically. Because the PIERCE technique creates a focal crack in the calcified plaque, its effectiveness may be limited when catheter fixation occurs over a broad or poorly localized calcified segment rather than at a discrete calcified point. Finally, a coaxial retrieval approach using the Wingman 35 was selected.

For the maneuvers, the entrapped microcatheter’s proximal hub is cut with a scalpel. The Wingman’s 0.035-inch inner lumen is sufficiently large to coaxially incorporate the stretched 0.014-inch-compatible microcatheter (outer diameter 2.1/2.8 Fr). Once successfully engaged, this strategy offers simultaneous plaque modification and vessel shielding. Because the entrapped system provides a stable coaxial track, it may help reduce the risk of vessel perforation by preventing the Wingman’s blade from deviating extravascularly.^7)^ In both presented cases, the Wingman 35 crossing catheter was used according to the same procedural concept: gentle coaxial advancement with a drilling motion at the calcified segment where the microcatheter was entrapped. Although the distance from the Wingman 35 tip to the microcatheter tip differed between cases, the device was manipulated at the site of entrapment within the calcified plaque. This maneuver likely modified the rigid calcified plaque and released the entrapped microcatheter. This technique may be broadly applicable to microcatheter entrapment within heavily calcified plaque; however, careful manipulation is mandatory because the entrapped catheter may already be stretched or structurally weakened. A relatively straight vascular course may facilitate Wingman advancement and reduce the risk of further catheter disruption. Once advanced over the lesion, the Wingman serves as a protective sheath, allowing the damaged system to be withdrawn safely without scraping the unraveled coil against the vessel wall. Finally, an antegrade approach is strongly recommended to maximize the Wingman’s linear penetration force and rotational maneuverability, which could be compromised in a crossover approach.^7)^ Although the Wingman may also be considered as a primary crossing device for heavily calcified lesions, conventional microcatheter systems supported by a diagnostic catheter remain advantageous for identifying residual microchannels because of their superior steerability. In addition, because the Wingman 35 is relatively expensive, costing approximately 179000 JPY in Japan, its routine first-line use may not be justified when conventional microcatheter-based crossing remains feasible. We therefore consider the Wingman 35 a useful additional option in selected situations, such as failure of conventional techniques, or when direct plaque modification is required to release an entrapped catheter. This technique is best suited for carefully selected cases. Appropriate anatomical characteristics include severely calcified lesions in which the microcatheter or guidewire remains partially disrupted but not completely fractured and in which the Wingman can be advanced antegradely in a coaxial fashion through a relatively straight vessel. Conversely, markedly tortuous vessels, complete catheter disruption, or an inability to advance the Wingman coaxially should be regarded as relative contraindications. Because the Wingman incorporates a needle tip, there remains a potential risk of guidewire, microcatheter, or vessel injury. To minimize these risks, the entrapped system should first be gently tensioned to obtain a linear configuration, and the Wingman should be advanced cautiously under continuous fluoroscopic observation. Because the Wingman 35 crossing catheter is available in effective lengths of up to 135 cm, the 235-cm Gladius guidewire used in the present cases was sufficiently long for coaxial advancement and manipulation of the device.

The main limitation of this report is that it is based on only 2 cases from a single center. Nevertheless, this technique may expand the endovascular bailout options for impending catheter fracture in severely calcified peripheral lesions. Further studies are required to evaluate its safety and efficacy across different microcatheters and lesion characteristics.

## Conclusion

Using a Wingman 35 crossing catheter as a coaxial retrieval tool may be a feasible bailout option for carefully selected cases of catheter entrapment with partial disruption in severely calcified peripheral lesions after failure of conventional retrieval techniques.

## Supplementary Materials

Supplementary Video 1 Fluoroscopic video showing the successful retrieval of the entrapped microcatheter in Case 1. A Wingman 35 crossing catheter is advanced coaxially over the locked system with a drilling motion through the calcified lesion, allowing successful retrieval of the entrapped microcatheter and microguidewire as a single unit.

Supplementary Video 2 Fluoroscopic video showing the successful retrieval of the entrapped microcatheter in Case 2. A Wingman 35 crossing catheter is advanced coaxially over the disrupted system by drilling through the calcified plaque, enabling the entrapped microcatheter and microguidewire to be pulled into the Wingman 35 and retrieved together.
